# Empiric Treatment of Classical Pyoderma Gangrenosum in the Outpatient Setting: A Case Report

**DOI:** 10.7759/cureus.62475

**Published:** 2024-06-16

**Authors:** Camille Basurto, Anthony Aponte-Diaz, Katharine Burmaster

**Affiliations:** 1 Internal Medicine, LewisGale Medical Center, Salem, USA

**Keywords:** pyoderma gangrenosum, outpatient care, leg ulcer, bacterial skin infection, chronic ulcer

## Abstract

Pyoderma gangrenosum (PG) is a rare neutrophilic disorder that typically presents as painful, ulcerative lesions. It is a diagnosis of exclusion and is oftentimes associated with systemic conditions such as inflammatory bowel disease, rheumatoid arthritis, and other inflammatory conditions. PG remains difficult to diagnose, and a delay in recognizing the disease can contribute to appreciable morbidity in the population. Here, we present the case of a 42-year-old male with the classical subtype of PG in the outpatient clinic who failed three courses of antibiotics before responding to corticosteroids.

## Introduction

Pyoderma gangrenosum (PG) is an autoimmune skin condition with an incidence of approximately 3-10 per one million every year [1,2]. Accurate diagnosis is paramount, as delay in treatment can contribute to increased morbidity in these patients [3,4]. Epidemiological evidence also illustrates that PG is associated with inflammatory bowel disease (IBD), rheumatoid arthritis, and solid or hematologic malignancies [5]. Thus, the workup of presumed PG should include the evaluation of systemic conditions and appropriate specialty referral and management [1]. Diagnosis of PG is made upon the exclusion of other skin conditions and is largely clinical in nature, misdiagnosis may occur, and thus, criteria have been established to ensure prompt and effective treatment [6-8]. The classical subtype of PG occurs on the lower extremities usually after trauma with a characteristic vesicle that ulcerates with rolled, purple, violaceous borders and surrounding erythema [1]. Pathologically, PG has a dense neutrophilic dermal infiltrate that is helpful but nonspecific. We present an outpatient case of a 42-year-old male who had an ulceration consistent with the classical subtype of PG. 

## Case presentation

A 42-year-old Caucasian male presented to the outpatient internal medicine clinic for hospital follow-up for one month of a solitary ulcerative wound with a violaceous border on his left anterior shin. Images were not captured at that time. The patient may have had trauma associated with the lesion, endorsed severe pain with movement, but denied systemic symptoms. He had been to the emergency department (ED) twice in the past two weeks due to this wound and was initially given a seven-day course of clindamycin 600 mg twice a day followed by seven-day courses of cephalexin 500 mg four times a day and doxycycline 100 mg twice a day. He was compliant with both antibiotic courses. Worsening erythema and cobblestoning prompted his second visit to the ED. Labs were largely unremarkable, notable only for mild leukocytosis (12,000 cells/microliters). Bedside ultrasound done at the ED showed the region was neurovascularly intact and hair was growing on the limb. At the first outpatient visit, he was instructed to continue antibiotic coverage, walk to improve circulation to the region, and do saltwater soaks. 

Two months later, the patient presented to the outpatient clinic for the same lesion. He states that initially the boil appeared to resolve when given antibiotics. Shortly afterward, however, a vesicle began to appear again and expand with scant sanguineous drainage. The pain was locally associated with the ulcerative lesion, now measuring 2×2 cm with surrounding 12×12 cm surrounding erythema. Similarly, the patient denied any fevers, chills, nausea, vomiting, diarrhea, and lymphadenopathy. At this outpatient visit, the boil appeared cellulitic in nature, and he was given repeat seven days of both cephalexin 500 mg four times a day and doxycycline 100 mg twice a day. A wound culture was obtained. 

**Figure 1:**
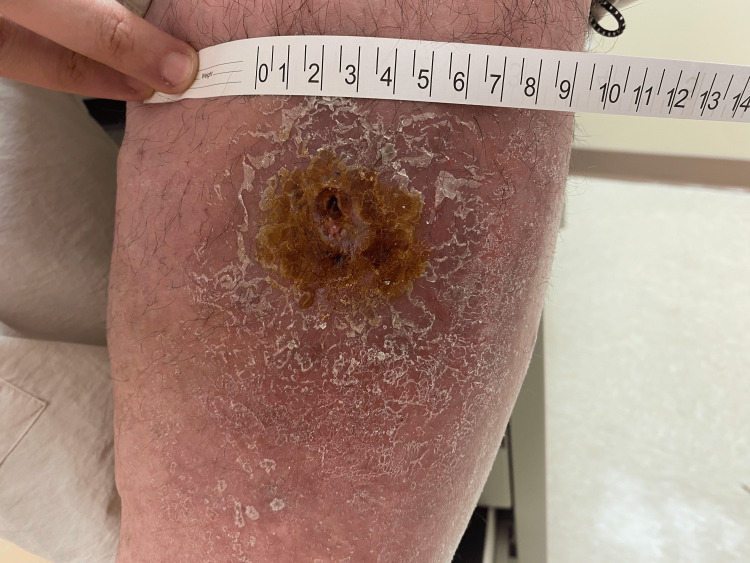
Left anterior shin erythematous ulceration with violaceous border measuring 2×2 cm with white scale and 9×9 cm of erythema without pustular discharge.

Two weeks later, the patient presented to the clinic after completing this third course of antibiotics. The wound was erythematous with central ulceration, measuring 2×2 cm with surrounding erythema 9×9 cm (Figure 1). Wound culture revealed no anaerobic growth. At this point in the patient's course, a nonbacterial and rather autoimmune cause for recurrent ulcerations was considered. Prednisone 60 mg daily for seven days was prescribed. 

**Figure 2:**
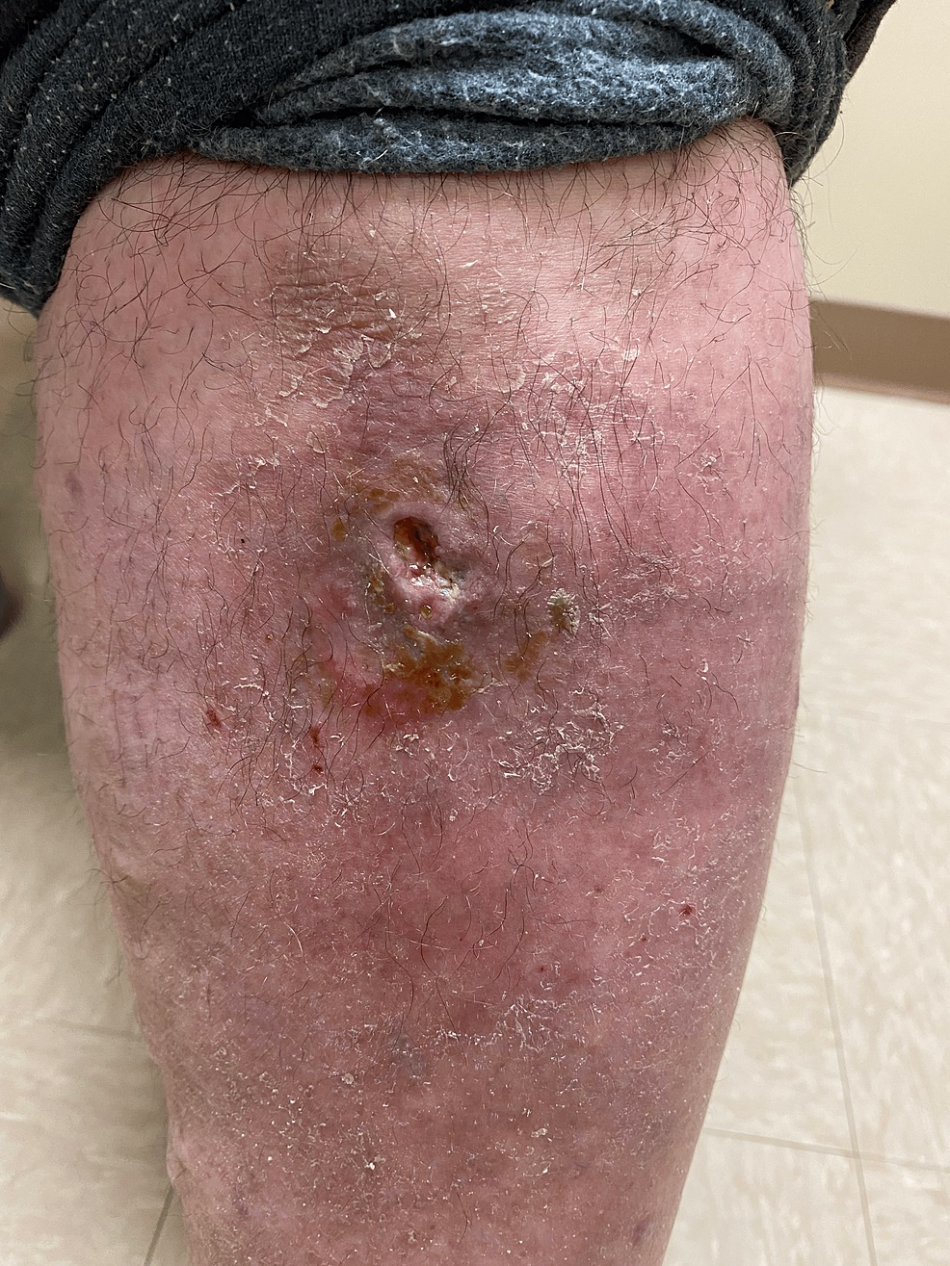
Following a seven-day course of prednisone, the same left anterior shin erythematous ulcerative lesion with violaceous border measuring 1.5×1.5 cm with 6×6 cm of erythema.

Two weeks later, the patient completed a seven-day course of prednisone. The wound had much improved, with less associated drainage (Figure 2). The ulcerative lesion 1.5×1.5 cm had 6×6 cm of erythema. He was prescribed a 10-day prednisone taper due to his response to the initial prednisone dosage, and a follow-up was scheduled. At follow-up four months later, the ulceration had resolved aside from residual skin pitting without further pain, erythema, or drainage.

## Discussion

PG is a rare autoimmune ulcerative skin condition that typically presents as a painful vesicle that breaks and expands into an ulceration [1]. It is diagnosed upon the exclusion of other ulcerative lesions and co-occurs with systemic conditions in up to 50% of cases [5]. Diagnosis of PG remains a clinical challenge and may be misdiagnosed [9]. Furthermore, PG has an up to three times increased risk of mortality when compared to the general population, and thus, efficacious treatment and appropriate referral remain imperative [3].

As PG is a diagnosis of exclusion, several criteria have been proposed to aid healthcare practitioners in reaching the diagnosis. Three such scoring criteria include the PARACELSUS criteria, Su et al.'s criteria, and the Delphi Consensus of International Experts criteria [6-8]. A cross-sectional study evaluating these three diagnostic criteria revealed that the PARACELSUS score had the highest proportion of confirmed diagnoses (approximately 90%) [10]. The PARACELSUS scoring system enumerates major, minor, and additional criteria most often seen in PG, and the summation of these factors delineates whether PG is highly likely or not. In our case, the patient scored an 11 prior to biopsy: major criteria including progressing disease (3 points), reddish-violaceous wound border (3 points), minor criteria including amelioration by immunosuppressive drugs (2 points), characteristically irregular (bizarre) ulcer shape (2 points), and additional criteria for undermined wound border (1 point). A score of 10 or greater makes PG highly likely. 

When evaluating ulcerations, the differential includes lesions seen in vasculitides (e.g., Wegener's granulomatosis), venous stasis ulcers, or acute cellulitis which may mimic PG [1,4]. Other considerations include livedoid vasculopathy, anti-phospholipid antibody syndrome lesions, and polyarteritis nodosa. Prior research by Weenig et al. found that in the 64 participants, overdiagnosis of PG occurred in up to 10% of lesions [11]. Therefore, a thorough workup with the examination of associated disorders and routine follow-up to assess the response of the disease prevents misdiagnosis. In our case, ruling out an associated infectious cause with wound culture, failure of multiple antibiotic courses, response to steroids at follow-up, and utilization of PARACELSUS screening criteria helped us arrive at our conclusion. 

Morphologically, this case provides an example of the classical subtype of PG of the lower extremities that responded to immunosuppressive treatment [1,4]. By treating PG empirically in the appropriate clinical scenario, we avoid skin biopsy that can worsen existing lesions. PG exhibits pathergy or the expansion of a lesion in the direction or pattern of trauma. Generally, patients should be told to avoid all surgery with PG, as it can lead to poor wound healing [12,13]. In our case, we encouraged close follow-up to the clinic. 

## Conclusions

PG is a rare skin condition with a poorly understood pathophysiology. Without prompt treatment, PG can further expand and cause local tissue damage. The diagnosis of PG may also be a harbinger of deeper pathological processes including solid or hematologic malignancies, IBD, or other autoimmune conditions. We illustrate the classical subtype of PG treated in the outpatient primary care setting to contribute to a greater understanding of this skin condition and its management. 
